# Nicorandil regulates the macrophage skewing and ameliorates myofibroblasts by inhibition of RhoA/Rho‐kinase signalling in infarcted rats

**DOI:** 10.1111/jcmm.13130

**Published:** 2017-11-09

**Authors:** Tsung‐Ming Lee, Shinn‐Zong Lin, Nen‐Chung Chang

**Affiliations:** ^1^ Cardiology Section Department of Medicine An‐Nan Hospital China Medical University Tainan Taiwan; ^2^ Department of Medicine China Medical University Taichung Taiwan; ^3^ Cardiovascular Research Laboratory China Medical University Hospital Taichung Taiwan; ^4^ Department of Internal Medicine School of Medicine College of Medicine Taipei Medical University Taipei Taiwan; ^5^ Department of Neurosurgery Tzu Chi University Hospital Hualien Taiwan; ^6^ Bioinnovation Center Tzu Chi foundation Hualien Taiwan; ^7^ Division of Cardiology Department of Internal Medicine Taipei Medical University Hospital Taipei Taiwan

**Keywords:** ATP‐sensitive potassium channel, interleukin‐10, M2 macrophage, myocardial infarction, myofibroblast

## Abstract

We have demonstrated that ATP‐sensitive potassium (K_ATP_) channel agonists attenuated fibrosis; however, the mechanism remained unclear. Since RhoA has been identified as a mediator of cardiac fibrosis, we sought to determine whether the anti‐fibrotic effects of K_ATP_ channel agonists were mediated *via* regulating macrophage phenotype and fibroblast differentiation by a RhoA/RhoA‐kinase‐dependent pathway. Wistar male rats after induction of myocardial infarction were randomized to either vehicle, nicorandil, an antagonist of K_ATP_ channel glibenclamide, an antagonist of ROCK fasudil, or a combination of nicorandil and glibenclamide or fasudil and glibenclamide starting 24 hrs after infarction. There were similar infarct sizes among the infarcted groups. At day 3 after infarction, post‐infarction was associated with increased RhoA/ROCK activation, which can be inhibited by administering nicorandil. Nicorandil significantly increased myocardial IL‐10 levels and the percentage of regulatory M2 macrophages assessed by immunohistochemical staining, Western blot, and RT‐PCR compared with vehicle. An IL‐10 receptor antibody increased myofibroblast infiltration compared with nicorandil alone. At day 28 after infarction, nicorandil was associated with attenuated cardiac fibrosis. These effects of nicorandil were functionally translated in improved echocardiographically derived cardiac performance. Fasudil showed similarly increased expression of M2 macrophages as nicorandil. The beneficial effects of nicorandil on fibroblast differentiation were blocked by adding glibenclamide. However, glibenclamide cannot abolish the attenuated fibrosis of fasudil, implying that RhoA/RhoA‐kinase is a downstream effector of K_ATP_ channel activation. Nicorandil polarized macrophages into M2 phenotype by inhibiting RhoA/RhoA‐kinase pathway, which leads to attenuated myofibroblast‐induced cardiac fibrosis after myocardial infarction.

## Introduction

Cardiac remodelling after myocardial infarction (MI) is a complex inflammation process involving numerous signalling pathways. Among the cells involved in mediating inflammation, macrophages and fibroblasts are two major players in tissue repair and fibrosis 1. Macrophages are a heterogeneous population of which the M1 and M2 macrophages represent the two most extreme subtypes 2. M1 macrophages have been implicated in tissue destruction, whereas M2 macrophages have been implicated in wound healing and tissue repair. The identification of M2 macrophages that can promote resolution of fibrotic disease is a relatively recent advance in the field of fibrotic research. A defining marker of regulatory macrophage function is the secretion of IL‐10 3. These macrophages secrete a variety of growth factors and cytokines, thereby modulating fibroblast activity and differentiating into myofibroblasts.

The other important cell type in myocardial healing and fibrosis, fibroblasts, differentiates into myofibroblasts and produce new extracellular matrix, mainly in the form of collagen. Myofibroblasts are key effectors of the tissue remodelling process. Fully differentiated myofibroblasts are characterized by the expression of α‐smooth muscle actin (α‐SMA). They possess contractile properties and are associated with a smaller and stronger scar area that helps to prevent infarct expansion and ventricular dilatation. However, the reactive fibrosis by myofibroblasts at the remote area contributes to the deterioration of left ventricular (LV) function 4. Thus, myofibroblasts can be targetted with beneficial clinical impact. Despite the relevance of macrophages and fibroblasts in tissue homeostasis, remarkably little is known whether macrophages are able to influence the properties of myofibroblasts. The identification of the relevant cell populations and a better understanding of the signals that regulate their anti‐inflammatory activity may lead to improved strategies for post‐infarction remodelling.

The molecular mechanisms for excessive infarction‐induced collagen synthesis after MI have not been fully clarified. RhoGTPase and its downstream target, Rho‐kinase (ROCK), may be an important therapeutic target for fibrotic diseases. RhoA is mainly localized in the cytosol in its RhoA‐guanosine diphosphate inactive form. The exchange from guanosine diphosphate to guanosine triphosphate on RhoA and its translocation to the membrane are markers of activation. RhoA has been directly implicated in regulating the formation of actin stress fibres, as evidenced by the induction of stress fibres and focal adhesions in fibroblasts upon microinjection of an activated mutant form of RhoA 5. Activation of Rho/ROCK promotes monomeric G‐actin polymerization into filamentous actin, resulting in nuclear import of myocardin‐related transcription factor‐A, a serum‐responsive factor co‐activator 6. In the nucleus, myocardin‐related transcription factor‐A binds serum‐responsive factor and activates fibrogenic gene programs that promote myofibroblast differentiation, collagen synthesis, and myofibroblast survival 7, 8, 9. Previous studies showed that the ROCK inhibitor fasudil can suppress cardiac fibrosis after MI 10. Furthermore, targetted deletion of ROCK protects the heart against pressure overload by inhibiting reactive fibrosis 11.

We previously demonstrated that activation of ATP‐sensitive potassium (K_ATP_) channel was associated with attenuated myocardial fibrosis after MI 12. However, the involved mechanisms remained unclear. Previous studies have shown that the peri‐conditioning stimulus of hydroxyfasudil, a ROCK inhibitor, was abrogated by glibenclamide, a non‐selective K_ATP_ channel blocker 13, and suggested that activation of K_ATP_ channels could be mediated by ROCK inhibition. The requirement for RhoA or its effectors, including ROCK, in K_ATP_ channel‐induced fibrosis, is unknown. RhoA plays a crucial role in the transduction of mechanical signals into cellular responses 14. Active RhoA induces actin polymerization, thereby inducing α‐SMA promoter activity 14. Furthermore, very recently, ROCK signalling has been shown to be as a master switch in macrophage polarization 15. We investigated the hypothesis that the activity of ROCK is involved in K_ATP_ channel‐induced cardioprotection by regulating the two M1/M2 macrophage phenotypes and myofibroblasts. The purpose of this study was (1) to investigate whether chronic administration of a K_ATP_ channel agonist, nicorandil, results in attenuated myocardial fibrosis through regulation of M2 and myofibroblast accumulation, and (2) to assess the role of RhoA/ROCK in cardiac fibrosis in a rat MI model using a ROCK inhibitor, fasudil (1‐(5‐isoquinolinesulfonyl)‐homopiperazine). In this study, we found that nicorandil administration alternatively modulates the macrophage phenotype and may contribute to cardiac repair.

## Methods

All rats received humane care and the experiment was approved and conducted in accordance with local institutional guidelines of the China Medical University for the care and use of laboratory animals and conformed with the National Institutes of Health *Guide for the Care and Use of Laboratory Animals*.

### Animals

#### Part 1

Male Wistar rats (200–250 g) were subjected to ligation of the left anterior descending artery as previously described 12, resulting in infarction of the LV free wall. Rats were randomly assigned into 6 groups so as to have approximately the same number of survivors in each group: (1) vehicle group; (2) nicorandil (0.1 mg/kg per day, Chugai Pharmaceutical Co., Japan), a specific mitochondrial K_ATP_ channel agonist; (3) glibenclamide (1.4 mg/kg per day), a nonspecific K_ATP_ channel blocker; (4) a combination of nicorandil and glibenclamide; (5) fasudil (10 mg/kg per day, Sigma‐Aldrich, St Louis, USA), a ROCK inhibitor; and (6) a combination of fasudil and glibenclamide. The doses of nicorandil 12 and glibenclamide 12 used in this study have been shown to specifically modulate K_ATP_ channels without the interference of haemodynamics. To verify the role of RhoA/ROCK in K_ATP_ channel inhibition‐related fibrosis, we further assessed fasudil in the role of ventricular remodelling. At higher concentrations, fasudil could inhibit other serine/threonine kinases, such as protein kinase A and protein kinase C 16. Thus, we used a low dose of fasudil 13, showing effective attenuation of cardiac fibrosis at a subhypertensive dose. The drugs were given orally by gastric gavage once a day. The drugs were started 24 hrs after MI, during which drugs can maximize benefits at this timing window 17 and minimize the possibility of a direct effect on infarct size. The heart was excised at days 3 or 28 after MI as early and late stages of MI. To prevent hypoglycaemic attacks during the administration of glibenclamide, 2.5% (W/V) sucrose in filtered tap water was supplied and glucose examinations were performed once per week by the one‐touch method. Sham operation served as controls to exclude the possibility of drugs themselves directly to alter fibrosis.

#### Part 2

Although results of the above study showed that nicorandil significantly increased M2 macrophage infiltration after infarction (see Results), the effect of increased M2‐induced IL‐10 levels on fibroblast activation remained unclear. We randomized the infarcted rats 24 hrs after inducing MI into vehicle, nicorandil (0.1 mg/kg per day) and the combination of nicorandil + anti‐IL‐10 R1. Anti‐IL‐10 R1 antibody (5 μg in 200 μl saline, BioLegend, San Diego, CA, USA) was given *via* intraperitoneal injection to block IL‐10 binding, once a day, for 3 days, starting at the first day after inducing infarction. At day 3 after infarction, all hearts (*n* = 7 per group) were used for Western blot of α‐SMA and for immunohistology of myofibroblast infiltration at the border zone (<2 mm outside the infarct).

### Experimental MI

After anaesthesia with intraperitoneal dose of ketamine‐xylazine (90–9 mg/kg), rats were intubated and the left anterior descending artery was ligated using a 6‐0 silk as our previous description 12. Sham rats underwent the same procedure except the suture was passed under the coronary artery and then removed. Mortality in the animals with MI was ~30% within the first 24 hrs. None of the sham‐operated animals died.

### Echocardiogram

At day 28 after operation, echocardiographic measurements were done under lightly anaesthesia with intraperitoneal injection of ketamine‐xylazine (25–4 mg/kg). For a detailed method, please refer to the Supplementary material online.

### Infarct size and haemodynamic measurements

A section, taken from the equator of the LV, was fixed in 10% formalin and embedded in paraffin for determination of infarct size. Haemodynamic parameters were measured at the end of the study as described in detail in the Supplementary material online.

### Real‐time RT‐PCR of IL‐6, IL‐1β, iNOS, CD206, and IL‐10

Real‐time quantitative RT‐PCR was performed from samples obtained from the border zone with the TaqMan system (Prism 7700 Sequence Detection System, PE Biosystems) at day 3 as previously described 12. We analysed the expression of gene markers for M1 (*IL‐6*,* IL‐1*β, *iNOS*) and M2 (*CD206*,* IL‐10*) macrophages. mRNAs were quantified by real‐time RT‐PCR with *cyclophilin* as a loading control. For a detailed method, please refer to the Supplementary material online.

### Western blot analysis of RhoA translocation, iNOS, IL‐10, and α‐SMA

Samples were obtained from either the border zone at day 3 or the remote zone (>2 mm outside the infarct) at day 28. Experiments were replicated three times and results expressed as the mean value as described in detail in the Supplementary material online.

### Immunohistochemical analysis of CD68, iNOS, IL‐10 and α‐SMA

To confirm the downstream pathways of K_ATP_ channel, immunohistochemical staining for M1 and M2 markers was performed on LV muscle. Cryosections were performed at a thickness of 5 μm and incubated with antibodies against CD68 (a marker for all macrophages; Abcam, Cambridge, MA, USA), iNOS (a marker for M1; Cell Signalling Technology, Danvers, MA, USA), IL‐10 (a marker for M2c; R& D systems, Abingdon, UK), and α‐SMA (a marker for myofibroblast; Sigma, St. Louis, MO, USA). The antibody had been tested for specificity in the rat. Isotype‐identical directly conjugated antibodies served as a negative control. Ten random scans per section were analysed and averaged. Quantification was calculated as the percentage of positively stained area to total area at a magnification of 400×.

### Morphology and morphometry of cardiac fibrosis

The interstitial collagen area fraction was determined by quantitative morphometry of the picrosirius‐stained sections. For a detailed method, please refer to the Supplementary material online.

### Laboratory measurements

Plasmin activity, a marker for inflammatory responses 18 as measured in serum (Roche, Applied Science, Indianapolis, IN, USA).

Histologic collagen results were confirmed by hydroxyproline assay adapted from Stegemann and Stalder 19. The samples from the remote zone were immediately placed in liquid nitrogen and stored at −80°C until measurement of the hydroxyproline content. The results were calculated as hydroxyproline content per weight of tissue.

Myocardial arginase activity assay for M2a and IL‐10 activity for M2c. Myocardial tissues from the border zones were homogenized in extraction buffer (50 mM potassium phosphate buffer, pH 7.0; 1 mM EDTA; 1 mM ethylene glycol tetraacetic acid; 0.2 mM phenylmethanesulfonylfluoride; 1 μg/ml pepstatin; 0.5 μg/ml leupeptin; 10 mM NaF; 2 mM Na_3_VO_4_; and 10 mM β‐mercaptoethanol), and centrifuged for 30 min. at 14,000 *g* at 4°C. 40 μg of each sample was mixed with 25 μl 500 mM L‐arginine (pH 9.7) in a 1.5 ml tube, incubated 1 hr at 37°C, then 400 μl acid (1:3:9, Sulphuric Acid: Phosphoric Acid: ddH_2_O) and 25 μl 9% ISPF in EtOH were added to each tube. Tubes were boiled for 45 min., then were determined at abs 550, which was calibrated against a standard curve of urea by colorimetric analysis (QuantiChrom Arginase Assay Kit, BioAssay Systems, Hayward, CA, USA). Arginase activity was expressed as a percentage of the vehicle group. Myocardial membrane‐bound IL‐10 fractions were measured using commercially available ELISA kits (R&D Systems).

### Statistical analysis

Results were presented as mean ± SD. Statistical analysis was performed using the SPSS statistical package (SPSS, version 18.0, Chicago, IL, USA). Differences among the groups of rats were tested by an ANOVA. In case of a significant effect, the measurements between the groups were compared with Bonferroni's correction. The significant level was assumed at value of *P* < 0.05.

## Results

### Part 1: acute stage (day 3)

Heart tissue was harvested at day 3 after MI. Differences in mortality and infarct size among the infarcted groups were not found at the acute stage of MI.

#### Nicorandil administration stimulates macrophages toward a M2 phenotype through RhoA/ROCK signalling

**Figure 1:**
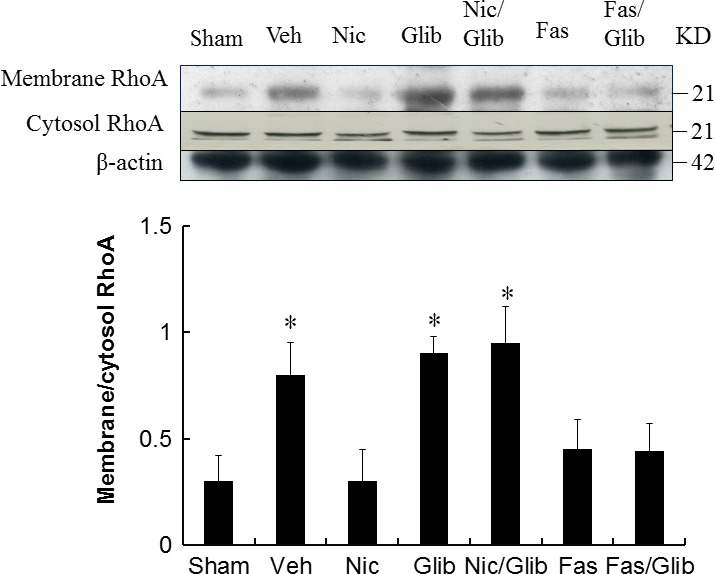
Western analysis of RhoA membrane fraction and cytosolic fraction from the border zone at day 3 after MI. Relative abundance was obtained by normalizing the protein density against that of β‐actin. Each column and bar represents mean ± SD. Each point is an average of 3 separate experiments. Sham (*n* = 10), Vehicle (Veh, *n* = 10), Nicorandil (Nic, *n* = 12), Glibenclamide (Glib, *n* = 11), Nic + Glib (*n* = 11), Fasudil (Fas, *n* = 9) and Fas + Glib (*n* = 9). *P < 0.05 compared with sham, Nic‐, Fas‐, and Fas + Glib‐treated groups. ANOVA with Bonferroni's correction.

The RhoA/ROCK signalling pathway is essential for the polarization of macrophages. We therefore examined the effect of nicorandil on RhoA/ROCK signalling by Western blot. The RhoA activity was studied in membrane and cytosolic protein preparations. MI increased membrane RhoA level by 2.67 folds (Fig. 1), consistent with activation of RhoA. Treatment with nicorandil decreased RhoA membrane level to 38 ± 12% of vehicle. However, nicorandil did not affect cytosol RhoA level compared with vehicle (*P* > 0.05, Fig. 1).

**Figure 2:**
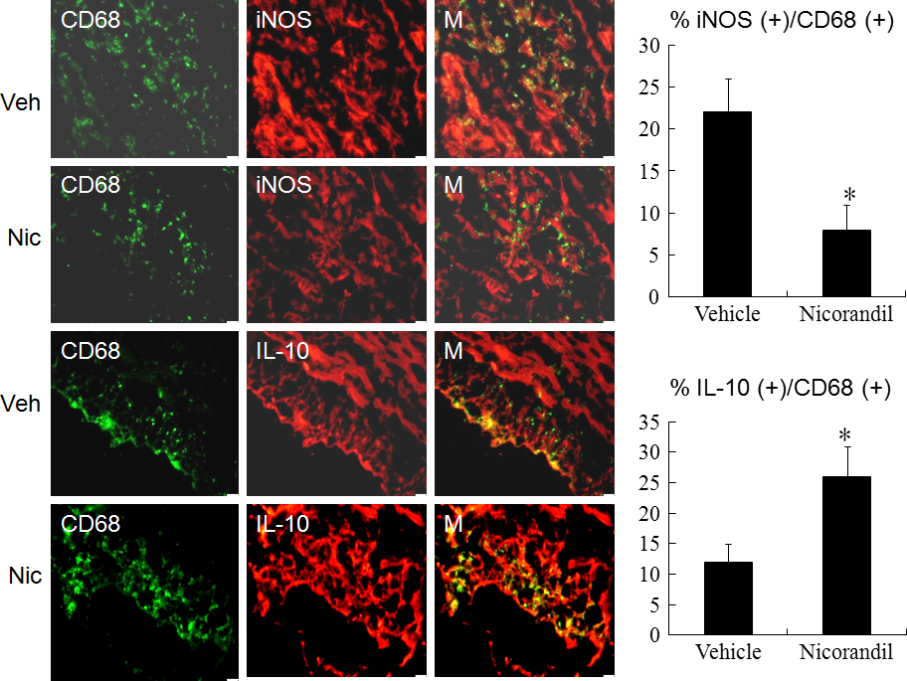
Immunohistochemical staining of M1 (CD68^+^, iNOS ^+^) and M2c (CD68^+^, IL‐10^+^) macrophage phenotype from the border zone at day 3 after MI. iNOS‐expressing CD68 (+) M1 macrophages were observed in infarcted myocardium treated with vehicle (Veh), but were significantly reduced by nicorandil (Nic) administration (upper panels). IL‐10‐expressing CD68 (+) M2 macrophages were predominant in nicorandil administered infarcted myocardium (lower panels). The iNOS‐expressing CD68 (+) or IL‐10‐expressing CD68 (+) macrophages were calculated and expressed as bar graphs. M, merged. The line length corresponds to 20 μm. Vehicle (Veh, *n* = 8), Nicorandil (Nic, *n* = 8). *P < 0.05 compared with vehicle.

Because macrophage phenotypic change plays a critical role in tissue repair and fibrosis, we characterized the effect of nicorandil on macrophage differentiation by examining type‐specific surface marker. Immunohistochemical staining demonstrated that CD68 (+) macrophages were infiltrated in the infarcted groups at day 3 after MI. To identify the subtype of infiltrated macrophages in infarcted myocardium, the marker for M1 (CD68^+^, iNOS^+^) and M2 (CD68^+^, IL‐10^+^) was examined in infarcted myocardium (Fig. 2). iNOS‐expressing CD68^+^ macrophages were observed in the vehicle (23 ± 4% in the vehicle *versus* 9 ± 3% in the nicorandil, *P* < 0.05), and IL‐10‐expressing CD68^+^ macrophages were more frequent in the nicorandil (12 ± 3% in the vehicle *versus* 27 ± 5% in the nicorandil group, *P* < 0.05). Significantly, nicorandil increased the percentage of M2c macrophages by 125% at 3 days after MI, compared with vehicle (Fig. 2; *P* < 0.01). Together, these data suggest improved ability of nicorandil‐treated rats to increase M2 macrophage differentiation with a concomitant reduction in M1.

**Figure 3:**
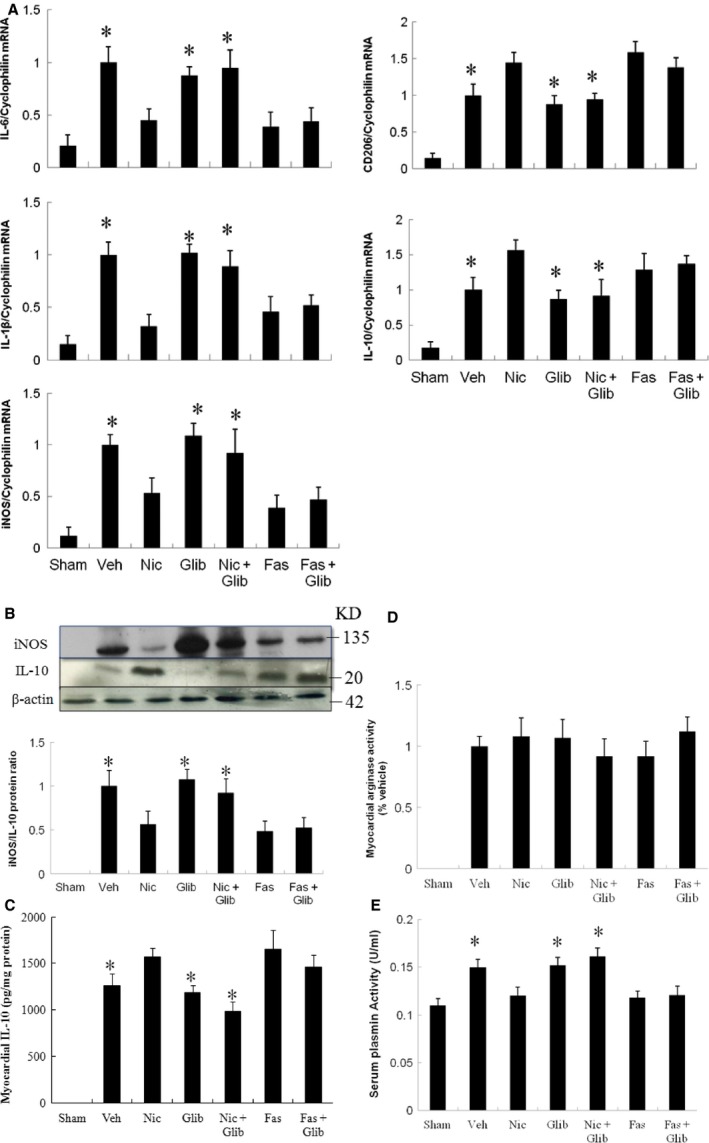
Expression of gene markers and function for M1 and M2 macrophages from the border zone at day 3 after MI. (A) mRNA, (B) protein levels, (C) myocardial IL‐10 activity, (D) myocardial arginase activity and (E) serum plasmin activity. There was a significant increase in macrophage accumulation in the infarcted heart at day 3 after MI. There is an obvious shift towards M2 macrophage phenotype in infarcted groups treated with nicorandil, fasudil and fasudil+ glibenclamide as shown by reduced M1 (*IL‐6*,*IL‐1*β, *iNOS*) expression, increased M2 (*CD206*,*IL‐10*) mRNA and protein, and attenuated plasmin activity. Sham (*n* = 10), Vehicle (Veh, *n* = 10), Nicorandil (Nic, *n* = 12), Glibenclamide (Glib, *n* = 11), Nic + Glib (*n* = 11), Fasudil (Fas, *n* = 9) and Fas + Glib (*n* = 9). *P < 0.05 compared with sham, Nic‐, Fas‐, and Fas + Glib‐treated groups. ANOVA with Bonferroni's correction.

Then, we analysed the expression and function of M1 and M2 macrophages. M1 (*IL‐6*,* IL‐1*β, *iNOS*) mRNA was remarkably decreased and M2 (*CD206, IL‐10*) mRNA was highly induced in the infarcted groups treated with nicorandil, fasudil and fasudil + glibenclamide (Fig. 3A). Then, we examined the protein levels of iNOS and IL‐10 by Western blotting (Fig. 3B). The IL‐10 level markedly increased in the nicorandil, fasudil and fasudil + glibenclamide groups. However, the increased IL‐10 level in the nicorandil group can be reduced after administering glibenclamide. The protein ratio of iNOS to IL‐10 was calculated and is shown in Figure 3B. These results showed that nicorandil administration leads to inhibitory responses on M1 markers and stimulatory effects on M2 markers.

Myocardial IL‐10 activity is shownin Figure 3C. IL‐10 activity levels were significantly higher (*P* < 0.05) in the nicorandil compared with vehicle. However, after adding glibenclamide, the IL‐10 activity was significantly decreased. Total arginase activity was similar among the infarcted groups (Fig. 3D).

Compared with vehicle, the plasmin activity (Fig. 3E) was decreased in the nicorandil, fasudil and fasudil + glibenclamide groups. The decreased plasmin level in the nicorandil group can be reversed after administering glibenclamide. However, the glibenclamide cannot reverse the plasmin levels in the fasudil group, implying the ROCK signalling is the downstream molecule of K_ATP_ channel.

#### Nicorandil‐induced M2 macrophages inhibit cardiac fibroblast activation *via* IL‐10 signalling

**Figure 4:**
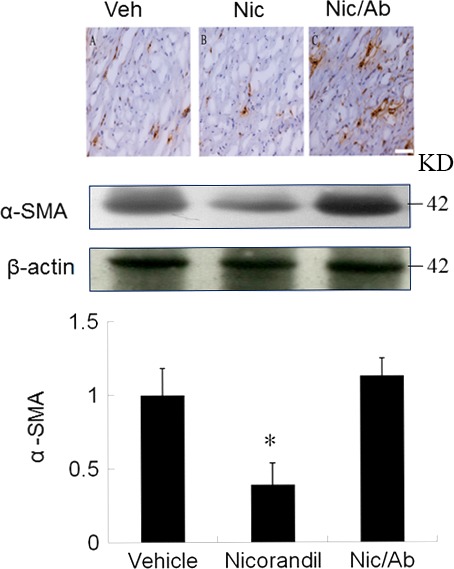
The role of IL‐10 on myofibroblast activation assessed by immunohistochemical staining and Western blot at day 3 after infarction. Positive staining for α‐SMA (brown color, a hallmark of myofibroblasts) in myocardium is significantly higher in the vehicle, which can be attenuated by administering nicorandil. After adding an IL‐10 receptor antibody (Ab), myofibroblast infiltration was significantly increased compared with nicorandil alone. Vehicle (Veh, *n* = 7), Nicorandil (Nic, *n* = 7), Nicorandil/IL‐10 receptor antibody (Nic/Ab, *n* = 7). *P < 0.05 compared with vehicle and Nic/Ab. ANOVA with Bonferroni's correction.

To determine whether nicorandil‐induced inhibition of myofibroblasts is through IL‐10, we added IL‐10 neutralizing antibody to block IL‐10. IL‐10 neutralizing antibody significantly decreased the expression of α‐SMA assessed by Western blot and immunohistochmistry (Fig. 4). Therefore, nicorandil‐induced M2 macrophages directly inhibited cardiac fibroblast differentiation into myofibroblasts through IL‐10 production.

### Part 2: chronic stage (day 28)

To assess the integrity of post‐infarction cardiac function, we examined echocardiography and histological sections 28 days after induced MI. Differences in mortality and infarct size among the infarcted groups were not found throughout the study. Nicorandil, glibenclamide and fasudil had little effect on cardiac gross morphology in the sham‐operated rats (data not shown). Four weeks after infarction, the infarcted area of the LV was very thin and was totally replaced by fully differentiated scar tissue. The vehicle‐treated infarcted group had an increase in right ventricular weight/body weight ratio and lung weight/body weight ratio, compared with nicorandil or fasudil‐treated infarcted groups. The weight of the LV inclusive of the septum remained essentially constant for 4 weeks among the infarcted groups (Table 1). Significant improvements of cardiac contractility (+dp/d*t*) and relaxation (−dp/d*t*) were observed in nicorandil‐treated infarcted rats compared with vehicle‐treated infarcted rats.

**Table 1:** Cardiac morphometry, haemodynamics, and echocardiographic findings at the end of study

Parameters	Sham	Infarction					
		Vehicle	Nic	Glib	Nic + Glib	Fasudil	Fas + Glib
No. of rats	10	10	12	11	11	9	9
Body weight, g	365 ± 25	372 ± 21	382 ± 21	377 ± 22	373 ± 20	392 ± 25	390 ± 27
HR, bpm	412 ± 23	394 ± 21	402 ± 18	419 ± 20	404 ± 16	415 ± 20	401 ± 18
LVESP, mm Hg	110 ± 8	98 ± 11	102 ± 7	103 ± 6	104 ± 8	96 ± 7	101 ± 8
LVEDP, mm Hg	6 ± 2	17 ± 3^a^	15 ± 3^a^	18 ± 4^a^	18 ± 3^a^	15 ± 3^a^	16 ± 5^a^
LVW/BW, mg/g	2.01 ± 0.11	3.13 ± 0.21^a^	2.89 ± 0.24^a^	3.22 ± 0.28^a^	3.05 ± 0.28^a^	2.82 ± 0.33^a^	3.22 ± 0.30^a^
RVW/BW, mg/g	0.45 ± 0.08	0.75 ± 0.10^a^	0.58 ± 0.07^a^, ^b^	0.82 ± 0.11^a^	0.76 ± 0.12^a^	0.54 ± 0.05^c^	0.85 ± 0.11^a^
LungW/BW, mg/g	4.21 ± 0.55	5.89 ± 0.53^a^	4.69 ± 0.49^b^	5.98 ± 0.33^a^	5.59 ± 0.65^a^	4.29 ± 0.49^c^	5.65 ± 0.35^a^
+dp/dt, mm Hg/sec.	7816 ± 342	3093 ± 325^a^	4250 ± 253^a^^,^ b	2834 ± 235^a^	2972 ± 264^a^	3882 ± 266^a^^,^ c	3063 ± 227^a^
‐dp/dt, mm Hg/sec.	5986 ± 278	2874 ± 327^a^	3377 ± 222^a^^,^ b	2762 ± 254^a^	2892 ± 253^a^	3291 ± 285^a^^,^ d	3026 ± 262^a^
Infarct size, %	…	40 ± 2	38 ± 3	41 ± 3	40 ± 2	40 ± 2	41 ± 3
LVEDD, mm	5.8 ± 0.2	8.6 ± 0.5^a^	7.8 ± 0.5^a^^,^ b	8.5 ± 0.4^a^	8.7 ± 0.5^a^	7.5 ± 0.4^a^^,^ c	8.4 ± 0.5^a^
LVESD, mm	3.6 ± 0.2	7.2 ± 0.3^a^	5.7 ± 0.4^a^^,^ b	7.1 ± 0.4^a^	7.2 ± 0.5^a^	5.4 ± 0.5^a^^,^ c	7.1 ± 0.3^a^
FS (%)	38 ± 2	16 ± 4^a^	27 ± 4^a^^,^ b	16 ± 3^a^	17 ± 4^a^	28 ± 4^a^^,^ c	15 ± 4^a^

Values are mean ± SD. BW: body weight; Fas: fasudil; FS: fractional shortening; Glib: glibenclamide; HR: heart rate; LungW: lung weight; LVEDD: left ventricular end‐diastolic dimension; LVEDP: left ventricular end‐diastolic pressure; LVESD: left ventricular end‐systolic dimension; LVESP: left ventricular end‐systolic pressure; LVW: left ventricular weight; Nic: nicorandil; RVW: right ventricular weight.

a *P* < 0.05 compared with sham.

b *P* < 0.05 compared with infarcted groups treated with vehicle and nicorandil + glibenclamide.

c *P* < 0.05 compared with infarcted groups treated with vehicle and fasudil + glibenclamide.

d *P* < 0.05 compared with vehicle‐treated infarcted group.

#### Echocardiographic data

**Figure 5:**
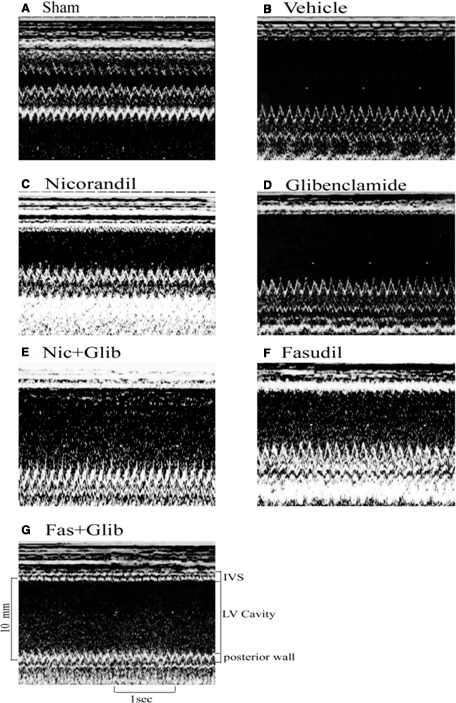
Representative M‐mode image reveals a hypokinetic‐to‐akinetic anterior wall and left ventricular (LV) dilation in the infarcted hearts (B–G) in contrast to normal anterior wall motion in sham‐operated heart (A). There are markedly dilated LV end‐diastolic diameter and LV end‐systolic diameter in groups treated with vehicle (B), Glibenclamide (D, Glib), Nicoradil (Nic) + Glib (E), and Fasudil (Fas) + Glib (G) compared with those in groups treated with Nic (C) and Fas (F). IVS, interventricular septum thickness.

Compared with sham‐operated hearts, MI hearts showed structural changes such as increased LV diastolic and systolic diameters (Table 1), consistent with LV remodelling. Both LVEDD and LVESD in rats with MI were significantly reduced by nicorandil or fasudil treatment (*P* < 0.0001, Fig. 5). LV fractional shortening was significantly higher in the nicorandil‐ or fasudil‐treated group compared with vehicle. Conversely, the rats to which glibenclamide was administered developed impaired LV systolic function and progressive LV dilation than that in the nicorandil‐ and fasudil‐treated groups alone. These data were corroborated by the results that +dP/d*t* was significantly improved in the nicorandil‐ and fasudil‐treated groups compared with the combined groups.

#### Effects of nicorandil and fasudil on myofibroblasts by immunohistochemical staining and Western blot at the chronic stage

**Figure 6:**
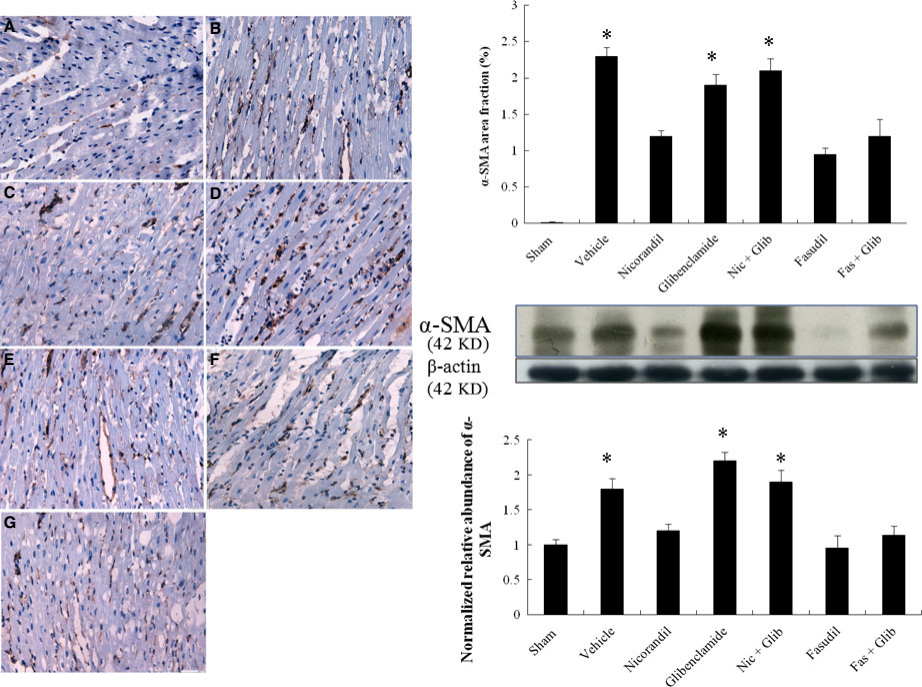
Myofibroblast infiltration at day 28 after infarction. Representative immunohistochemical staining of α‐SMA, a hallmark of myofibroblasts (magnification 400×) at the remote zone in different treated rats. A, sham; B, vehicle; C, nicorandil; D, glibenclamide; E, nicorandil + glibenclamide; F, fasudil; G, fasudil + glibenclamide. The line length corresponds to 50 μm. To further confirm the protein levels of α‐SMA, Western blot was performed, showing similar results of immunohistochemical staining. Each column and bar represents mean ± SD. Sham (*n* = 10), Vehicle (Veh, *n* = 10), Nicorandil (Nic, *n* = 12), Glibenclamide (Glib, *n* = 11), Nic + Glib (*n* = 11), Fasudil (Fas, *n* = 9) and Fas + Glib (*n* = 9). *P < 0.05 compared with sham, Nic‐, Fas‐, and Fas + Glib‐treated groups. ANOVA with Bonferroni's correction.

Immunohistochemical analysis of the infarcted myocardium revealed the presence of α‐SMA–expressing myofibroblasts in the myocardial tissue at 4 weeks after infarction (Fig. 6A). Stronger α‐SMA signals at the remote zone of vehicle‐treated rats were observed than those in the same region of sham rats. The intensity of the immunoreaction was reduced in the nicorandil‐, fasudil‐ and fasudil + glibenclamide‐treated groups compared with that in the vehicle. To further confirm the findings of immunohistochemical staining, Western blot was performed by using an antibody specific for myofibroblasts, α‐SMA. Similarly, the levels of α‐SMA were significantly reduced in the nicorandil‐, fasudil‐ and fasudil + glibenclamide‐treated groups compared with that in the vehicle (Fig. 6B). However, nicorandil‐induced beneficial effects were reversed by the addition of glibenclamide, implicating K_ATP_ channels as the relevant target.

#### Effects of nicorandil and fasudil on remote myocardial fibrosis

**Figure 7:**
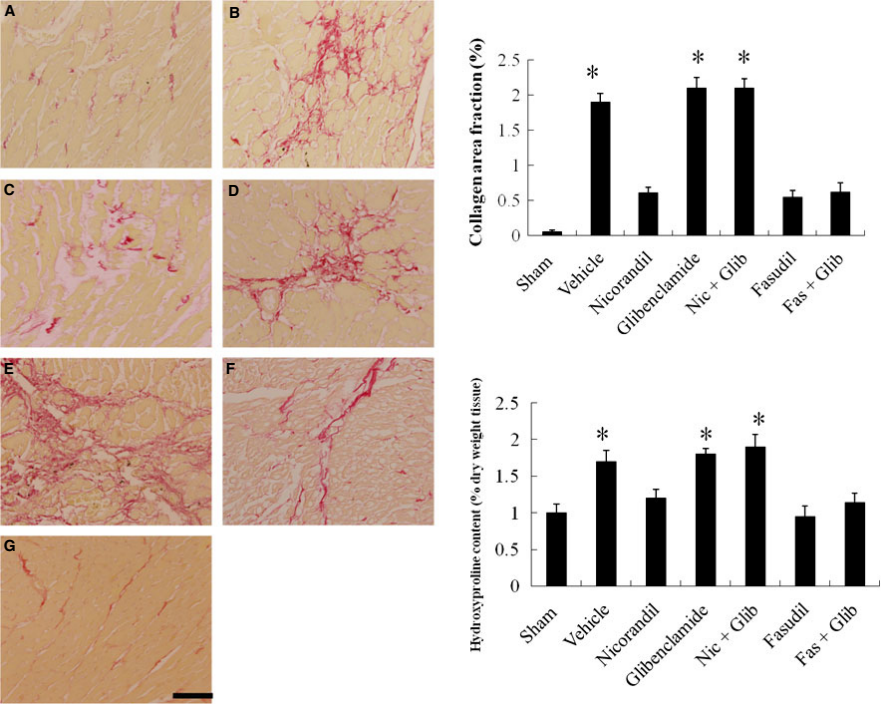
Cardiac fibrosis at the remote zone at day 28. Representative sections from the remote zone with Sirius Red staining (red, magnification 400×) at 4 weeks after infarction. The line length corresponds to 50 μm. Hydroxyproline content was also shown to measure quantitative amount of fibrosis. Each column and bar represents mean ± SD. Sham (*n* = 10), Vehicle (Veh, *n* = 10), Nicorandil (Nic, *n* = 12), Glibenclamide (Glib, *n* = 11), Nic + Glib (*n* = 11), Fasudil (Fas, *n* = 9) and Fas + Glib (*n* = 9). *P < 0.05 compared with sham, Nic‐, Fas‐, and Fas + Glib‐treated groups. ANOVA with Bonferroni's correction.

Fibrosis of the LV from the remote zone was examined in tissue sections after Sirius Red staining as shown in Figure 7. Infarcted rats treated with vehicle had significantly larger areas of intense focal fibrosis compared with sham‐operated rats (1.90 ± 0.12% *versus* 0.06 ± 0.02%, *P* < 0.0001). Compared with vehicle, treatment with nicorandil, fasudil, or fasudil + glibenclamide attenuated fibrosis. Collagen formation in the LV was significantly increased in rats treated with a combination of nicorandil and glibenclamide compared with nicorandil‐treated rats alone.

Hydroxyproline content was measured (Fig. 7). There was a marked increase in collagen content after MI, and this was significantly attenuated by either nicorandil or fasudil. Surprisingly, hydroxyproline content in the LV similarly changed in rats treated with a combination of fasudil and glibenclamide compared with fasudil‐treated rats alone.

## Discussions

In this study, we demonstrate that nicorandil inhibited RhoA translocation and inactivates ROCK, which enhanced M2 macrophage activation, resulting in reduced infiltration of myofibroblast and collagen accumulation at the remote zone. Our results provide novel insight into a macrophage‐mediated mechanism of cardioprotection following ischaemic injury. Our results were consistent with the beneficial effects of nicorandil, as documented structurally by increase in M2 infiltration and reduction in cardiac fibrosis, molecularly by myocardial iNOS and IL‐10 protein and mRNA levels, biochemically by tissue hydroxyproline levels, and functionally by improvement of echocardiographically derived fractional shortening. Distinctive changes in macrophage gene expression and function underlie the cardioprotective effects of nicorandil. These studies identify a novel anti‐fibrotic role of nicorandil involving inhibition of myofibroblasts and suggest that targetting myofibroblasts with K_ATP_ channel agonists may be of therapeutic benefit after infarction.

**Figure 8:**
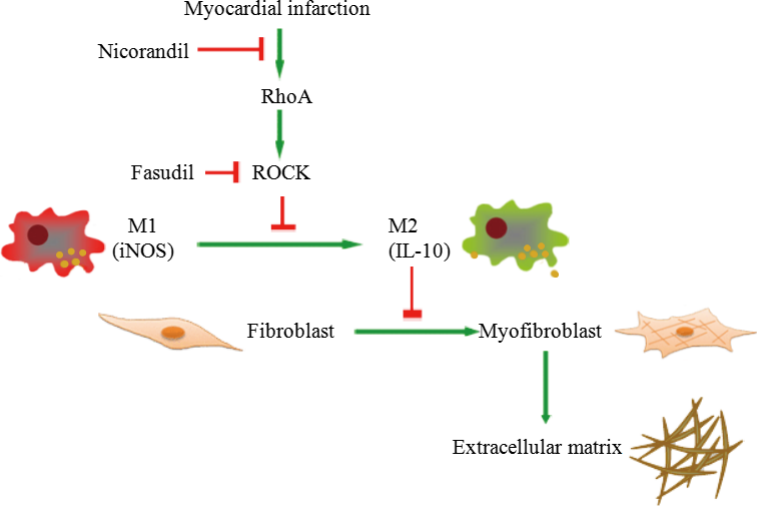
Proposed schematic representation illustrates the involvement of nicorandil in macrophage‐ and myofibroblast‐related cardiac fibrosis in post‐infarcted rats. After MI, during the early stage, it is characterized by inflammatory M1 phenotype macrophages, however, at a late stage, M2 phenotype would become dominant. M1 and M2 phenotype macrophages can be converted into each other. IL‐10 activity in M2 macrophages inhibits myofibroblast differentiation, which antagonizes extracellular matrix production. Nicorandil administration exhibits potent anti‐fibrotic activity by suppressing collagen synthesis *via* increasing the activation of M2 macrophages and inhibiting myofibroblast differentiation.

This heterogeneity of macrophages in injured tissues relates to different drugs that also undergo changes during the different phases of dynamic disease processes, which then is associated with shifts toward different macrophage populations. Although both M1 and M2 activation are rapidly induced following MI, M2 activation has been shown to be late and transient in the MI 20. Our results showed improved heart function by shifting M2 macrophages, consistent with the findings that M2 depletion was associated with a loss in left ventricular contractile function 21. Therefore, facilitating M2 activation is a promising strategy for promoting tissue repair *via* inflammatory responses following MI. The effect of nicorandil on macrophage subpopulation and attenuated cardiac fibrosis was supported by 3 lines of evidence (Fig. 8).

1. Administration of K_ATP_ channel agonists attenuates the dysfunction of chronically infarcted hearts. Cardiac fibrosis and echocardiographically derived LVESD and LVEDD were significantly smaller in nicorandil‐treated infarcted group than those in the vehicle. The beneficial effects of nicorandil were abolished after adding glibenclamide, indicating that these effects were achieved *via* the activation of K_ATP_ channel. Our results were consistent with the findings of Coromilas *et al*. 22, showing that K_ATP_ channels remain functional after infarction and can be activated by K_ATP_ channel agonists.
2. Nicorandil can modify macrophages from an M1 to an M2 phenotype through a ROCK‐dependent pathway. The result was consistent with recent studies, showing that ROCK signalling can modulate polarization of macrophages 15, 23. Studies both *in vivo* and *in vitro* have demonstrated that macrophages can undergo dynamic transitions between the M1 and M2 states of activation 24, 25. Despite these recent advances, the signalling pathways involved in regulating the macrophage polarization from the M1 to M2 phenotype are poorly understood. Infarction was associated with increased RhoA/ROCK activation as shown by enhanced RhoA translocalization from the cytosol to the membrane. A significant decrease in RhoA content in the cytosol fraction was not detected in our study, most likely because of the relative abundance of RhoA in the cytosol fraction. ROCK, one of the downstream targets of RhoA, has been shown to be involved in a variety of actions of Rho. To determine whether ROCK was involved in the RhoA induced macrophage phenotypes, the rats were treated with a ROCK inhibitor, fasudil. Fasudil polarizes M1 into M2 macrophages after MI, an effect similar to that of nicorandil. The results were consistent with the findings of Kushiyama *et al*. 26, showing that fasudil shifted M1 to M2 macrophages in experimental renal disease. Our study showed that glibenclamide abrogated the effect of nicorandil on RhoA activity, indicating that K_ATP_ channel activation modulates RhoA activity. Furthermore, fasudil was still able to attenuate ROCK activity in rats treated with the antagonist of K_ATP_ channels glibenclamide, suggesting that the ROCK is the downstream effector of K_ATP_ channels.
3. Fibroblast transdifferentiation was regulated by IL‐10 activity. Previous studies have shown that M2 macrophages can modulate the differentiation of fibroblasts into myofibroblasts 27. Previous studies have shown that M2 played a role in inhibiting, rather than promoting, fibroblast transdifferentiation at the remodelling phase 28. Furthermore, we extended previous studies and showed that M2 macrophages are able to inhibit myofibroblast differentiation *via* production of M2‐producing IL‐10. The reduced myofibroblast differentiation after administering nicorandil was consistent with the decrease of plasmin activity. Indeed, our results were consistent with the findings of Minami *et al*. 29, showing that decreased plasmin activity is associated with reduced cardiac fibrosis.

To assess the role of IL‐10 in the regulation of fibroblast transdifferentiation, infarcted rats treated with an IL‐10 receptor antibody, had significantly increased myofibroblast infiltration, implying the role of IL‐10 in fibroblast transdifferentiation. Thus, it is not surprising to know that IL‐10‐expressing macrophages impede extracellular matrix deposition in fibrotic diseases 30. M2 macrophages contribute to the control of inflammatory process through the release of IL‐10 and arginase, a process which promotes controlled wound healing and tissue regeneration. M2 macrophages have been shown to include three well recognized sub‐populations (M2a, M2b, and M2c), each with its own distinct inducers, markers, and functions. The M2a phenotype is characterized by high expression of arginase‐1 and the M2c phenotype, IL‐10 31. The M2a macrophages are known to contribute to the formation of extracellular matrix through the upregulation of arginase levels, which were similar among the infarcted groups in our study. Our study showed that nicorandil preferentially activated M2c assessed by increased IL‐10 expression and protein, rather than M2a evidenced by arginase activity. The result was consistent with the notion that unlike M2a macrophages, M2c macrophages can promote resolution of fibrotic disease by locally producing suppressor cytokines, including IL‐10 3.

### Other mechanisms

Although this study suggests that the mechanisms of a K_ATP_ channel agonist‐induced transition from M1 to M2 may be related to attenuated ROCK activity, other potential mechanisms need to be studied. Nicorandil has been shown to provide gastroprotection by increasing mucosa PGE_2_ 32. PGE_2_ has been suggested to play a role in the induction of IL‐10 in macrophages and switched from M1 to M2 33. Thus, we cannot exclude the possible role of nicorandil‐induced PGE_2_ in macrophage polarization. A more extensive array analysis would be required to determine the hierarchy of M2 regulation. It is likely that several signalling pathways act together to operate the tight regulation of macrophage skewing at the time of resolution of inflammation.

Interestingly, although there was similar cardiac fibrosis between the infarcted rats treated with fasudil and with fasudil/glibenclamide, we found that the ratio of right ventricular weight/body weight was significantly higher in the rats treated with fasudil/glibenclamide compared with those treated with fasudil. Given RhoA/ROCK signalling is one of cardiac hypertrophy 34, fasudil attenuated right ventricular hypertrophy by inhibiting RhoA/ROCK signalling. Besides RhoA/ROCK signalling, K_ATP_ channels regulate a number of other signalling pathways. We have previously demonstrated that glibenclamide increased cardiomyocyte hypertrophy *via* activated p70S6 kinase 35, which cannot be affected by adding fasudil. Thus, it is not surprising that the rats co‐treated with glibenclamide had higher right ventricular weight than that in fasudil alone.

### Clinical implications

The study suggests that adequate adjustment of cardiac macrophage polarity and macrophage heterogeneity under pathological conditions can be cardioprotective. Nicorandil, initially characterized as a K_ATP_ channel agonist, has been administered to patients with coronary artery disease. In this study, we have extended the effect of nicorandil to attenuate inflammatory cell infiltration in the myocardium. Proper macrophage‐mediated transitions through the phases of repair are influenced by injury severity, age, health of the individual. However, if phenotypic characterization of M2 macrophages has not been performed or delayed to work, persistence of myofibroblasts potentiates a prolonged inflammatory phase and remodelling is not properly initiated. Nicorandil shifts M1 to M2 macrophage phenotype, producing less fibroblast transdifferentiation. The anti‐fibrotic effect of nicorandil is an additive beneficial action that contributes to promoting cardiac performance. Besides, M2 macrophages‐associated cytokines may support the growth of resident cardiac stem cells, which play a role in cardiac regeneration after MI while pro‐inflammatory (M1 macrophages)‐associated cytokines inhibit the growth of resident cardiac stem cells 36. Furthermore, in the atherosclerosis plaque, the increased M2 infiltration was associated with plaque regression and prevented plaque rupture 37. Thus, nicorandil‐induced M2 macrophages may improve cardiac function by attenuated fibrosis, increased survival of resident cardiac stem cells, and stabilized vulnerable plaque.

After acute MI, patients remain at high risk for recurrent cardiovascular events and mortality 38. Myofibroblasts are responsible for the production and deposition of collagen, leading to the establishment of a dense extracellular matrix that strengthens the infarcted tissue and minimizes dilatation of the infarct area. However, fibrosis might extend to remote zone and cause adverse remodelling of the cardiac tissue. This eventually leads to the development of congestive heart failure. Novel therapeutic agents targetting myofibroblasts are being developed to successfully prevent the cardiac remodelling of sites remote from the infarct area and therefore hinder the establishment of heart failure. Thus, the K_ATP_ channel agonists may have important biological effects that prevent occurrence of post‐infarcted heart failure.

Besides, apart from contributing to fibrotic remodelling, myofibroblasts induce arrhythmogenic slow conduction and ectopic activity in cardiomyocytes after establishment of heterocellular electrotonic coupling *in vitro*. After ablation of α‐SMA containing stress fibres, myofibroblasts lose their arrhythmic effects on cardiomyocytes, even if heterocellular electrotonic coupling is sustained 39. Thus, ROCK inhibitors might be useful for treating cardiovascular diseases in humans, in addition to the current indication of cerebral vasospasm, including angina pectoris, hypertension, pulmonary hypertension, stroke and heart failure. A specific delineation of the ways in which inflammation‐ and ischemia‐driven signals impact the fibrotic remodelling may have important implications for the treatment of a variety of ischaemic disorders.

### Study limitations

There are limitations in the translation of the results of this study to other species and to human physiology. First, recently, M2 activation was shown to be facilitated in resident microglia but not in infiltrating macrophages after spinal cord injury 40. Resident tissue macrophages have limited self‐renewal capacity during steady conditions and are replenished from the pool of circulating monocytes. Following MI, endogenous cardiac‐derived chemotactic signals and danger‐associated molecular patterns are released from the infarcted tissue, promoting the expansion of resident and monocyte‐derived macrophages into distinct phenotypes. The present study did not investigate the origin of polarized macrophages. However, Heidt *et al*. 41 have shown almost complete disappearance of resident cardiac macrophages in the first 24 hrs after MI. Thus, the anti‐inflammatory macrophages may stem from the infiltrating macrophages. Second, despite the beneficial effect of fasudil on LV remodelling, overall mortality was unchanged. Thus, a longer period of observation would be needed to evaluate the prognostic effect of fasudil in the present model. Third, polarized macrophages can theoretically be distinguished by their different receptor expression profiles, as well as differential cytokine production. However, polarized macrophages retain their plasticity to respond to environmental signals. Therefore, a single biochemical marker to discriminate macrophage populations remains difficult, and multiple markers are typically examined to distinguish macrophage phenotypes. At present, there is no single specific marker that can accurately discriminate between these sub‐populations. In this study, we differentiated the macrophage subtypes by immunohistochemical staining, Western blot, and RT‐PCR. Finally, nicorandil is a hybrid compound of K_ATP_ channel opener and nitrate action. The possibility cannot be ruled out that the nitrate action of nicorandil may also play a role in attenuating myocardial fibrosis. Glibenclamide has been shown to act as both a K_ATP_ channel blocker and a vasorelaxant 42. Glibenclamide induces NO generation, which is primarily responsible for the glibenclamide‐induced endothelium‐dependent relaxation 43. If the beneficial effect of nicorandil on attenuated myocardial fibrosis was explained by NO rise, the rats treated with glibenclamide should be expected to have similar myocardial fibrosis compared with nicorandil alone. Obviously, this was not the case. The effect of nicorandil on attenuated myocardial fibrosis was abolished by treatment with glibenclamide, further confirming the predominant role of K_ATP_ channels in this phenomenon.

In conclusion, the preferential shift of the macrophage phenotype from M1 to M2 may be related to K_ATP_ channel agonists‐related inhibition of the ROCK pathway that contributes to cardiac fibrosis. Our findings also demonstrate how nicorandil attenuates myocardial fibrosis through the down‐regulation of RhoA by targetting M2 macrophages. The study provides novel insight into to mechanisms of nicorandil as a potential antifibrogenic agent in prevention and treatment of cardiac fibrosis after MI.

## Conflict of Interest

None declared.

## Funding

This work was supported by the grants of An‐Nan Hospital (ANHRF 105‐01) and Ministry of Science and Technology (MOST 104‐2314‐B‐039‐021 and MOST 105‐2314‐B‐039‐042), Taiwan.

T.M.L. and N.C.C. performed the research; T.M.L., S.Z.L. and N.C.C. designed the research study; T.M.L. and S.Z.L. analysed the data; T.M.L. wrote the paper.

## Supporting information


**Appendix S1** Method.
