# Supplementary material for: Nicorandil regulates the macrophage skewing and ameliorates myofibroblasts by inhibition of RhoA/Rho‐kinase signalling in infarcted rats
Source: J Cell Mol Med. 2017 Nov 9;22(2):1056–69. doi: 10.1111/jcmm.13130 (PMC5783972; doi:10.1111/jcmm.13130)
Supplement: Supplementary file 1 — Appendix S1 Method. [file JCMM-22-1056-s001.docx]

**Supplemental Method**

### Echocardiogram

At day 28 after operation, rats were lightly anesthetized with intraperitoneal injection of ketamine-xylazine (25 mg/kg-4 mg/kg). Echocardiographic measurements were done using the GE Healthcare Vivid 7 Ultra-sound System (Milwaukee, WI) equipped with a 14-MHz probe as previously described (1). M-mode tracing of the LV was obtained from the parasternal long-axis view to measure LV end-diastolic diameter dimension (LVEDD) and LV end-systolic diameter dimension (LVESD), and fractional shortening (FS) (%) was calculated. After this, the hearts quickly underwent hemodynamic measurement after systemic heparinization.

##### *Hemodynamics and Infarct size measurements*

Hemodynamic parameters were measured in anesthetized rats with an additional intraperitoneal dose of ketamine-xylazine (90 mg/kg-9 mg/kg) at the end of the echocardiogram. A polyethylene Millar catheter was inserted into the LV and connected to a transducer (Model SPR-407, Miller Instruments, Houston, TX) to measure LV systolic and diastolic pressure as the mean of measurements of five consecutive pressure cycles as previously described (1). The maximal rate of LV pressure rise (+dP/d*t*) and decrease (-dP/d*t*) was measured. After the arterial pressure measurement, the atria and the right ventricle were trimmed off, and the LV was rinsed in cold physiological saline, weighed, and immediately frozen in liquid nitrogen after obtaining a coronal section of the LV for infarct size estimation. A section, taken from the equator of the LV, was fixed in 10% formalin and embedded in paraffin for determination of infarct size. Each section was stained with hematoxylin and eosin, and trichrome. The infarct size was determined as previously described (2). With respect to clinical importance, only rats with large infarction (>30%) were selected for analysis.

***Real-time RT-PCR of IL-6, IL-1β, iNOS,CD206, and IL-10***

Real-time quantitative RT-PCR was performed from samples obtained from the border zone with the TaqMan system (Prism 7700 Sequence Detection System, PE Biosystems) at day 3 as previously described (1). We analyzed the expression of gene markers for M1 (*IL-6, IL-1β, iNOS*) and M2 (*CD206, IL-10*) macrophages. Primers sequences were the following:

*IL-6* sense 5'- CCAGTTGCCTTCTTGGGACTGATG-3', antisense 5'-ATTTTCTGACCACAGTGAGGAATG-3';

*IL-1β* sense 5'-ATGGCAACTGTCCCTGAACTCAACT-3', antisense 5'-CAGGACAGGTATAGATTCAACCCCTT-3';

*iNOS* sense 5'-TCACCTTCGAGGGCAGCCGA-3', antisense 5'-TCCGTGGCAAAGCGAGCCAG-3';

*CD206* sense 5'-TGGGTTTGCTGAAGAAGAGAA-3', antisense 5'-CATGTGATAAGTGACAAATGCTTG-3';

*IL-10* sense 5'-GGTTGCCAAGCCTTGTCAGAA-3', antisense 5'-GCTCCACTGCCTTGCTTTTATT-3';

*cyclophilin* sense 5’-ATGGTCAACCCCACCGTGTTCTTCG-3’, antisense 5’-CGTGTGAAGTCACCACCCTGACACA-3’.

Standard curves were plotted with the threshold cycles versus log template quantities. After initial denaturation, amplification was performed at 95°C (10 s) 60°C (5 s) 72°C (10 s) for 45 cycles. Fold change was normalized against *cyclophilin*, a housekeeping gene.

***Western Blot Analysis of RhoA translocation, iNOS, IL-10, and α-SMA***

Samples were obtained from either the border zone (<2 mm outside the infarct) at day 3 or the remote zone (>2 mm outside the infarct) at day 28. After spinning for 10 min, 1,000*g* at 4°C to remove nuclei, the supernatant was transferred into ultracentrifuge tubes and spun for 45 min at 100,000*g* at 4°C. The resulting pellet of membrane proteins and the supernatant cytosolic fraction were stored at −80°C. Membrane and cytosolic proteins were separated on SDS–PAGE. After incubation with antibodies against Rho (Santa Cruz Biotechnology Inc., Santa Cruz, CA), iNOS (Cell Signaling Technology, Danvers, MA, USA), IL-10 (R& D systems, Abingdon, UK), α-SMA (Clone 1A4, Sigma, St. Louis, Missouri), and β-actin (Santa Cruz Biotechnology, Santa Cruz, CA), the nitrocellulose membrane was then rinsed with a blocking solution and incubated for 2 hours at room temperature. Antigen-antibody complexes were detected with 5-bromo-4-chloro-3-indolyl-phosphate and nitroblue tetrazolium chloride (Sigma). Films were volume-integrated within the linear range of the exposure using a scanning densitometer. Experiments were replicated three times and results expressed as the mean value.

***Morphology and morphometry of cardiac fibrosis***

Coronal sections of the remote zone were prepared for light microscopic evaluation. Aniline blue and picrosirius, a collagen-specific stain (Sirius Red F3BA; Pfaltz & Bauer, Stamford, CT), was used to stain 5-µm thick, paraffin-embedded sections. The interstitial collagen area fraction was determined by quantitative morphometry of the picrosirius-stained sections with an automated image analyzer (Image Pro Plus, CA). These parameters were assessed in a blinded fashion by at least two investigators. The density of labeled areas were qualitatively estimated from 10 randomly selected fields at a magnification of 400×. The value was expressed as the ratio of labeled area to total area.

***Morphology and morphometry of cardiac fibrosis***

Coronal sections of the remote zone were prepared for light microscopic evaluation. Aniline blue and picrosirius, a collagen-specific stain (Sirius Red F3BA; Pfaltz & Bauer, Stamford, CT), was used to stain 5-µm thick, paraffin-embedded sections. The interstitial collagen area fraction was determined by quantitative morphometry of the picrosirius-stained sections with an automated image analyzer (Image Pro Plus, CA). These parameters were assessed in a blinded fashion by at least two investigators. The density of labeled areas were qualitatively estimated from 10 randomly selected fields at a magnification of 400×. The value was expressed as the ratio of labeled area to total area.

**Supplementary references**

1. Lee TM, Lin MS, Chang NC. [Effect of ATP-sensitive potassium channel agonists on ventricular remodeling in healed rat infarcts.](http://www.ncbi.nlm.nih.gov/pubmed/18371564) J Am Coll Cardiol. 2008;51:1309-1318.

2. Pfeffer MA, Braunwald E (1990) Ventricular remodeling after myocardial infarction. Circulation 81:1161–1172.
